# Supplementary material for: Lack of long-term acclimation in Antarctic encrusting species suggests vulnerability to warming
Source: Nat Commun. 2019 Jul 29;10:3383. doi: 10.1038/s41467-019-11348-w (PMC6662708; doi:10.1038/s41467-019-11348-w)
Supplement: Supplementary file 1 — Supplementary Information [file 41467_2019_11348_MOESM1_ESM.pdf]

Supplementary Information

Lack of long-term acclimation in Antarctic encrusting species suggests vulnerability to warming

Clark et al

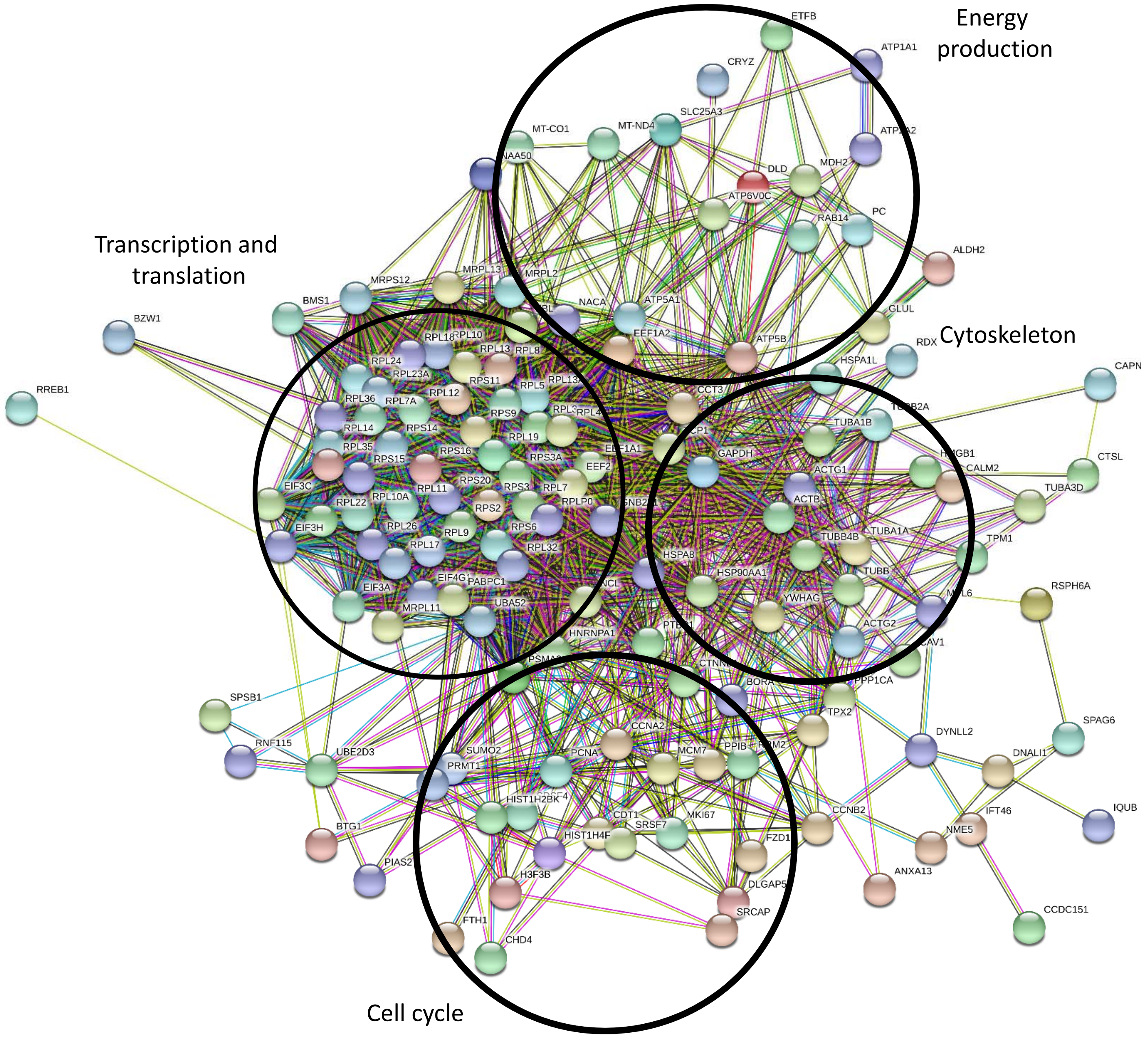

Supplementary Figure 1: STRING[1,2] output annotated with functional clusters

Supplementary Table 1: Transcriptome assembly and annotation metrics

**Assembly metrics**		
Total trinity genes	45,527	
Total trinity transcripts	61,421	
Total bases	29,824,840	
N50	713	
Contigs in N50	9,766	
Percentage GC	44	
Mean length for contigs	486	
Max contig length	49,432	
**Annotation metrics**		
	**Unique**	**Total**
gene_id	45,527	45,527
transcript_id	61,421	61,421
prot_id	14,658	14,658
prot_coords	8,266	14,658
sprot_Top_BLASTX_hit	13,915	13,919
sprot_Top_BLASTP_hit	9,045	9,301
Pfam	7,887	8,151
gene_ontology_pfam	695	4,972
TmHMM	999	1,029
SignalP	457	521

Supplementary Table 2: Enriched GO processes in the control versus +2°C transcriptome comparisons. Direction of "down" means that the processes were less represented in controls compared with +2 and thus effectively enriched in +2 compared to control samples.

**GO Biological Process**						
	No of Genes	Proportion Down	Proportion Up	Direction	P-Value	FDR
GO:0015031 PROTEIN_TRANSPORT	10	0.60	0.00	Down	0.001	0.011
GO:0006351 TRANSCRIPTION_DNA-TEMPLATED	29	0.59	0.14	Down	0.001	0.011
**GO Molecular Function**						
GO:0019843 RRNA_BINDING	24	0.75	0.00	Down	0.001	0.016
GO:0003899 DNA-DIRECTED_5'-3'_RNA_POLYMERASE_ACTIVITY	22	0.73	0.09	Down	0.001	0.016

Supplementary Table 3: Gene ontology and BLAST results for Trinity transcripts associated with Trinity genes with significant SNP results

NB: Each Trinity gene is often represented by several Trinity transcripts
NB: Not all SPROT annotations produced GO annotation. Gene Ontology (GO) terms represent molecular function, but if not available biological process (BP) is annotatec

Gene	Mean z-score	FDR	Number of SNPs	Transcript_id	SPROT_Top BLASTX_ID	SPROT_Top BLASTX_Gene	E-Value	gene_ontology_pfam	Gene notes
TRINITY_DN718458_c5_g4	0.49893653846	0	8	TRINITY_DN718458_c5_g4_i1			.	.	
TRINITY_DN648545_c0_g2	0.479467765568571	0.01	14	TRINITY_DN648545_c0_g2_i1			.	.	.
TRINITY_DN676495_c5_g3	0.498937	0.02	7	TRINITY_DN676495_c5_g3_i1			.	.	.
TRINITY_DN619912_c4_g2	0.4562551282045	0.02	20	TRINITY_DN619912_c4_g2_i6			.	.	
TRINITY_DN632889_c2_g1	0.452429059830667	0.02	15	TRINITY_DN632889_c2_g1_i16			.	.	.
TRINITY_DN648447_c1_g1	0.440471794873333	0.02	6	TRINITY_DN648447_c1_g1_i1			.	.	
TRINITY_DN629739_c11_g3	0.450740384615	0.02	8	TRINITY_DN629739_c11_g3_i2			.	.	
TRINITY_DN617264_c4_g1	0.445473767259231	0.02	13	TRINITY_DN617264_c4_g1_i5			.	.	
TRINITY_DN635787_c6_g1	0.445773504275	0.02	6	TRINITY_DN635787_c6_g1_i2			.	.	.
TRINITY_DN642498_c3_g5	0.447133936650588	0.02	17	TRINITY_DN642498_c3_g5_i3			.	.	
TRINITY_DN644365_c4_g5	0.456446153846923	0.02	13	TRINITY_DN644365_c4_g5_i1			.	.	.
TRINITY_DN666119_c2_g1	0.43596	0.025	17	TRINITY_DN666119_c2_g1_i1			.	.	.
TRINITY_DN666119_c2_g1	0.43596	0.025	17	TRINITY_DN666119_c2_g1_i2			.	.	.
TRINITY_DN666119_c2_g1	0.43596	0.025	17	TRINITY_DN666119_c2_g1_i3			.	.	.
TRINITY_DN743030_c21_g1	0.435082352941176	0.025	17	TRINITY_DN743030_c21_g1_i11	Q488Z6	RS5_COLP3	1.97E-50	.	Translation
TRINITY_DN743030_c21_g1	0.435082352941176	0.025	17	TRINITY_DN743030_c21_g1_i1	Q488Z6	RS5_COLP3	1.81E-99	GO:0003735: structural constituent of ribosome	Translation
TRINITY_DN743030_c21_g1	0.435082352941176	0.025	17	TRINITY_DN743030_c21_g1_i9	A1S233	RL6_SHEAM	1.26E-92	GO:0003735: structural constituent of ribosome	Translation
TRINITY_DN738280_c3_g1	0.431108717948	0.0257142857142858	10	TRINITY_DN738280_c3_g1_i12			.	.	.
TRINITY_DN738280_c3_g1	0.431108717948	0.0257142857142858	10	TRINITY_DN738280_c3_g1_i2	Q7PPA5	ATC1_ANOGA	9.71E-125	.	ATPase
TRINITY_DN637768_c7_g1	0.428149450549624	0.0293333333333334	21	TRINITY_DN637768_c7_g1_i28			.	.	
TRINITY_DN718236_c1_g2	0.424650641025	0.03875	8	TRINITY_DN718236_c1_g2_i1	A8WYE4	PAR1_CAEBR	5.21E-30	.	Cell division
TRINITY_DN611044_c4_g5	0.40194757834737	0.0470588235294118	27	TRINITY_DN611044_c4_g5_i1			.	.	
TRINITY_DN698079_c5_g3	0.39829179487125	0.0470588235294118	20	TRINITY_DN698079_c5_g3_i1			.	.	
TRINITY_DN649896_c4_g1	0.388163003663143	0.0470588235294118	14	TRINITY_DN649896_c4_g1_i5			.	.	
TRINITY_DN742681_c7_g2	0.38902104001951	0.0470588235294118	107	TRINITY_DN742681_c7_g2_i5	Q487Z8	RL22_COLP3	6.18E-72	GO:0003735: structural constituent of ribosome	Translation
TRINITY_DN624905_c4_g1	0.403066422466667	0.0470588235294118	42	TRINITY_DN624905_c4_g1_i20			.	.	
TRINITY_DN741750_c2_g1	0.385220512820146	0.0470588235294118	41	TRINITY_DN741750_c2_g1_i7	Q489A1	RL5_COLP3	4.08E-91	GO:0003735: structural constituent of ribosome	Translation
TRINITY_DN741750_c2_g1	0.385220512820146	0.0470588235294118	41	TRINITY_DN741750_c2_g1_i12	Q489A1	RL5_COLP3	7.88E-93	.	Translation
TRINITY_DN741750_c2_g1	0.385220512820146	0.0470588235294118	41	TRINITY_DN741750_c2_g1_i15	Q489A1	RL5_COLP3	3.17E-93	.	Translation
TRINITY_DN707721_c5_g2	0.38297	0.047059	38	TRINITY_DN707721_c5_g2_i1			.	.	.
TRINITY_DN707721_c5_g2	0.38297	0.047059	38	TRINITY_DN707721_c5_g2_i2			.	.	.
TRINITY_DN622435_c3_g1	0.38166866	0.047059	18	TRINITY_DN622435_c3_g1_i1			.	.	.
TRINITY_DN677665_c6_g5	0.391235294118471	0.0470588235294118	17	TRINITY_DN677665_c6_g5_i1			.	.	
TRINITY_DN673029_c7_g1	0.399041880341667	0.0470588235294118	18	TRINITY_DN673029_c7_g1_i8			.	.	
TRINITY_DN630680_c3_g1	0.373471794872727	0.0470588235294118	11	TRINITY_DN630680_c3_g1_i9	Q488Z7	RL18_COLP3	1.92E-32	.	Translation
TRINITY_DN630680_c3_g1	0.373471794872727	0.0470588235294118	11	TRINITY_DN630680_c3_g1_i8	Q488Z6	RS5_COLP3	2.73E-68	GO:0003723: RNA binding	Translation
TRINITY_DN630414_c5_g5	0.408940792540909	0.0470588235294118	11	TRINITY_DN630414_c5_g5_i16			.	.	.
TRINITY_DN700175_c3_g1	0.379014529914333	0.0470588235294118	30	TRINITY_DN700175_c3_g1_i22			.	.	
TRINITY_DN742681_c7_g1	0.3873480769225	0.0470588235294118	8	TRINITY_DN742681_c7_g1_i4	Q488A0	RL16_COLP3	4.62E-48	.	Translation
TRINITY_DN505940_c0_g1	0.387303625110345	0.0470588235294118	29	TRINITY_DN505940_c0_g1_i1			.	.	.
TRINITY_DN624293_c0_g2	0.40676307692396	0.0470588235294118	25	TRINITY_DN624293_c0_g2_i16			.	.	
TRINITY_DN640287_c2_g5	0.4020487179495	0.0470588235294118	20	TRINITY_DN640287_c2_g5_i2			.	.	
TRINITY_DN679445_c4_g1	0.401535519125164	0.0470588235294118	61	TRINITY_DN679445_c4_g1_i11			.	.	
TRINITY_DN647460_c6_g2	0.376043589743312	0.0470588235294118	16	TRINITY_DN647460_c6_g2_i3			.	.	
TRINITY_DN698009_c4_g1	0.384511396011333	0.0470588235294118	18	TRINITY_DN698009_c4_g1_i4			.	.	
TRINITY_DN672550_c4_g3	0.406298290598611	0.0470588235294118	36	TRINITY_DN672550_c4_g3_i15			.	.	.
TRINITY_DN672793_c4_g2	0.388730364373053	0.0470588235294118	19	TRINITY_DN672793_c4_g2_i8			.	.	
TRINITY_DN623316_c5_g1	0.398013333332	0.0470588235294118	5	TRINITY_DN623316_c5_g1_i2			.	.	
TRINITY_DN670777_c2_g1	0.372486303940463	0.0470588235294118	41	TRINITY_DN670777_c2_g1_i5	Q3BAI2	YCX91_PHAAO	9.44E-06	.	Uncharacterised
TRINITY_DN670777_c2_g1	0.372486303940463	0.0470588235294118	41	TRINITY_DN670777_c2_g1_i11			.	.	
TRINITY_DN635514_c2_g2	0.417911111111667	0.0470588235294118	12	TRINITY_DN635514_c2_g2_i8			.	.	.
TRINITY_DN671964_c10_g1	0.40625210256476	0.0470588235294118	25	TRINITY_DN671964_c10_g1_i6			.	.	.
TRINITY_DN628092_c0_g9	0.418957692307143	0.0470588235294118	28	TRINITY_DN628092_c0_g9_i3			.	.	
TRINITY_DN657568_c4_g2	0.386367881241653	0.0470588235294118	19	TRINITY_DN657568_c4_g2_i2			.	.	
TRINITY_DN622954_c10_g1	0.375572364672833	0.0470588235294118	18	TRINITY_DN622954_c10_g1_i3			.	.	.
TRINITY_DN670483_c5_g3	0.37897573483461	0.0470588235294118	41	TRINITY_DN670483_c5_g3_i1			.	.	
TRINITY_DN727351_c4_g2	0.402700197237923	0.0470588235294118	13	TRINITY_DN727351_c4_g2_i3			.	.	
TRINITY_DN617520_c5_g2	0.387002564102917	0.0470588235294118	30	TRINITY_DN617520_c5_g2_i24			.	.	.
TRINITY_DN640665_c1_g2	0.410135531135714	0.0470588235294118	14	TRINITY_DN640665_c1_g2_i4	Q487Z6	RL2_COLP3	2.38E-64	GO:0003735: structural constituent of ribosome	Translation
TRINITY_DN625486_c7_g1	0.39573846153875	0.0470588235294118	12	TRINITY_DN625486_c7_g1_i11	P02552	TBA1_CHICK	4.41E-133	.	Tubulin, cytoskeleton
TRINITY_DN625486_c7_g1	0.39573846153875	0.0470588235294118	12	TRINITY_DN625486_c7_g1_i3	P02552	TBA1_CHICK	2.02E-117	.	Tubulin, cytoskeleton
TRINITY_DN625486_c7_g1	0.39573846153875	0.0470588235294118	12	TRINITY_DN625486_c7_g1_i1	P02552	TBA1_CHICK	1.06E-143	.	Tubulin, cytoskeleton
TRINITY_DN706515_c1_g1	0.416898534798786	0.0470588235294118	14	TRINITY_DN706515_c1_g1_i19	Q05974	RAB1A_LYMST	2.17E-86	.	.
TRINITY_DN729286_c1_g6	0.373114245014222	0.0470588235294118	36	TRINITY_DN729286_c1_g6_i1			.	.	
TRINITY_DN652405_c3_g2	0.392371509971981	0.0470588235294118	54	TRINITY_DN652405_c3_g2_i21			.	.	.
TRINITY_DN619578_c3_g3	0.419376689976364	0.0470588235294118	22	TRINITY_DN619578_c3_g3_i10			.	.	
TRINITY_DN602732_c7_g1	0.400187933635588	0.0470588235294118	17	TRINITY_DN602732_c7_g1_i2			.	.	.
TRINITY_DN741429_c4_g1	0.392512820513077	0.0470588235294118	13	TRINITY_DN741429_c4_g1_i9	Q489T9	RS18_COLP3	3.1E-38	.	Translation
TRINITY_DN646956_c0_g2	0.410215042734533	0.0470588235294118	15	TRINITY_DN646956_c0_g2_i7			.	.	.
TRINITY_DN673722_c3_g2	0.418179975579048	0.0470588235294118	21	TRINITY_DN673722_c3_g2_i14			.	.	
TRINITY_DN619912_c4_g6	0.395285057471345	0.0470588235294118	29	TRINITY_DN619912_c4_g6_i2			.	.	.
TRINITY_DN704469_c1_g1	0.388023589744	0.0470588235294118	5	TRINITY_DN704469_c1_g1_i4			.	.	
TRINITY_DN634403_c6_g6	0.385922507123111	0.0470588235294118	18	TRINITY_DN634403_c6_g6_i1			.	.	
TRINITY_DN712909_c5_g1	0.382051092117778	0.0470588235294118	27	TRINITY_DN712909_c5_g1_i12	Q487Z7	RS19_COLP3	5.48E-33	.	Translation
TRINITY_DN712909_c5_g1	0.382051092117778	0.0470588235294118	27	TRINITY_DN712909_c5_g1_i23	Q487Z6	RL2_COLP3	3.38E-47	.	Translation
TRINITY_DN712909_c5_g1	0.382051092117778	0.0470588235294118	27	TRINITY_DN712909_c5_g1_i10	Q487Z6	RL2_COLP3	1.28E-55	.	Translation
TRINITY_DN712909_c5_g1	0.382051092117778	0.0470588235294118	27	TRINITY_DN712909_c5_g1_i1	Q487Z6	RL2_COLP3	5.47E-121	GO:0003735: structural constituent of ribosome	Translation
TRINITY_DN712909_c5_g1	0.382051092117778	0.0470588235294118	27	TRINITY_DN712909_c5_g1_i2	Q487Z7	RS19_COLP3	2.53E-27	.	Translation
TRINITY_DN712909_c5_g1	0.382051092117778	0.0470588235294118	27	TRINITY_DN712909_c5_g1_i14	Q487Z6	RL2_COLP3	9.39E-115	GO:0003735: structural constituent of ribosome	Translation
TRINITY_DN655853_c2_g2	0.378051676528461	0.0470588235294118	13	TRINITY_DN655853_c2_g2_i8			.	.	
TRINITY_DN617797_c4_g3	0.41645320512875	0.0470588235294118	16	TRINITY_DN617797_c4_g3_i2			.	.	
TRINITY_DN674687_c5_g4	0.381205128204667	0.0470588235294118	24	TRINITY_DN674687_c5_g4_i1			.	.	.
TRINITY_DN666119_c0_g1	0.40969839743625	0.0470588235294118	16	TRINITY_DN666119_c0_g1_i1			.	.	
TRINITY_DN668861_c7_g3	0.40083782051375	0.0470588235294118	8	TRINITY_DN668861_c7_g3_i1			.	.	.
TRINITY_DN742580_c3_g1	0.399635164835714	0.0470588235294118	7	TRINITY_DN742580_c3_g1_i8	Q47UV5	RL11_COLP3	2.22E-93	GO:0003735: structural constituent of ribosome	Translation
TRINITY_DN742580_c3_g1	0.399635164835714	0.0470588235294118	7	TRINITY_DN742580_c3_g1_i11	Q47UV5	RL11_COLP3	2.22E-98	GO:0003735: structural constituent of ribosome	Translation
TRINITY_DN700907_c9_g1	0.4000235897435	0.0470588235294118	20	TRINITY_DN700907_c9_g1_i3			.	.	.
TRINITY_DN685825_c8_g1	0.390132730015	0.0470588235294118	17	TRINITY_DN685825_c8_g1_i6			.	.	
TRINITY_DN635911_c5_g2	0.384263636363045	0.0470588235294118	22	TRINITY_DN635911_c5_g2_i1			.	.	
TRINITY_DN609892_c5_g4	0.377348368298477	0.0470588235294118	44	TRINITY_DN609892_c5_g4_i5			.	.	.
TRINITY_DN731884_c3_g7	0.400985347986286	0.0470588235294118	14	TRINITY_DN731884_c3_g7_i1			.	.	
TRINITY_DN636702_c5_g1	0.40527179487	0.0470588235294118	7	TRINITY_DN636702_c5_g1_i1			.	.	.
TRINITY_DN638801_c3_g1	0.403697094016667	0.0470588235294118	15	TRINITY_DN638801_c3_g1_i32			.	.	
TRINITY_DN454068_c0_g1	0.376427692308	0.0470588235294118	5	TRINITY_DN454068_c0_g1_i1			.	.	.
TRINITY_DN655312_c1_g3	0.39634045584	0.0470588235294118	18	TRINITY_DN655312_c1_g3_i1			.	.	
TRINITY_DN690907_c0_g1	0.388730355666129	0.0470588235294118	31	TRINITY_DN690907_c0_g1_i14	Q5QXV7	RS4_IDILO	1.94E-128	GO:0019843: rRNA binding	translation
TRINITY_DN690907_c0_g1	0.388730355666129	0.0470588235294118	31	TRINITY_DN690907_c0_g1_i28	Q488Z0	RS11_COLP3	2.83E-54	GO:0003735: structural constituent of ribosome	translation
TRINITY_DN690907_c0_g1	0.388730355666129	0.0470588235294118	31	TRINITY_DN690907_c0_g1_i9	Q488Z0	RS11_COLP3	3.73E-57	GO:0003735: structural constituent of ribosome	translation
TRINITY_DN690907_c0_g1	0.388730355666129	0.0470588235294118	31	TRINITY_DN690907_c0_g1_i1	Q488Z0	RS11_COLP3	1.04E-64	GO:0003735: structural constituent of ribosome	translation
TRINITY_DN728669_c1_g1	0.40562615384585	0.0470588235294118	20	TRINITY_DN728669_c1_g1_i8	Q488Z6	RS5_COLP3	1.7E-66	GO:0003735: structural constituent of ribosome	Translation
TRINITY_DN728669_c1_g1	0.40562615384585	0.0470588235294118	20	TRINITY_DN728669_c1_g1_i7	Q488Z6	RS5_COLP3	1.48E-66	GO:0003723: RNA binding	Translation
TRINITY_DN612873_c0_g1	0.391101831502857	0.0470588235294118	7	TRINITY_DN612873_c0_g1_i26	Q47UV7	RL10_COLP3	1.35E-63	GO:0042254: BP: ribosome biogenesis	Translation
TRINITY_DN612873_c0_g1	0.391101831502857	0.0470588235294118	7	TRINITY_DN612873_c0_g1_i9	Q47UV7	RL10_COLP3	5.15E-75	GO:0042254: BP: ribosome biogenesis	Translation
TRINITY_DN695745_c0_g1	0.371443589743917	0.0470588235294118	12	TRINITY_DN695745_c0_g1_i3	Q9VI93	RN_DROME	6.12E-35	.	Transcription factor
TRINITY_DN695745_c0_g1	0.371443589743917	0.0470588235294118	12	TRINITY_DN695745_c0_g1_i2	Q9VI93	RN_DROME	8.65E-62	.	Transcription factor
TRINITY_DN673071_c1_g2	0.389997435897333	0.0470588235294118	12	TRINITY_DN673071_c1_g2_i6	Q0P5A2	COQ5_BOVIN	2.77E-88	GO:0007017: BP: microtubule-based process	Transferase
TRINITY_DN673071_c1_g2	0.389997435897333	0.0470588235294118	12	TRINITY_DN673071_c1_g2_i10	Q0P5A2	COQ5_BOVIN	2.66E-65	.	Transferase
TRINITY_DN658689_c1_g2	0.41420512820625	0.0470588235294118	8	TRINITY_DN658689_c1_g2_i5			.	.	
TRINITY_DN697899_c0_g2	0.400646828610105	0.0470588235294118	38	TRINITY_DN697899_c0_g2_i9	Q6P0B1	CELF2_DANRE	1.03E-58	GO:0003676: nucleic acid binding	Translation
TRINITY_DN697899_c0_g2	0.400646828610105	0.0470588235294118	38	TRINITY_DN697899_c0_g2_i4	Q6P0B1	CELF2_DANRE	8.18E-60	GO:0003676: nucleic acid binding	Translation
TRINITY_DN661195_c5_g2	0.379856410256	0.0470588235294118	5	TRINITY_DN661195_c5_g2_i10			.	.	.
TRINITY_DN637856_c1_g1	0.375624615384258	0.0470588235294118	20	TRINITY_DN637856_c1_g1_i1			.	.	
TRINITY_DN670774_c1_g1	0.384197633136154	0.0470588235294118	13	TRINITY_DN670774_c1_g1_i20	P83510	TNIK_MOUSE	0	GO:0004672: protein kinase activity	Signalling
TRINITY_DN670774_c1_g1	0.384197633136154	0.0470588235294118	13	TRINITY_DN670774_c1_g1_i27	P83510	TNIK_MOUSE	9.51E-96	GO:0004672: protein kinase activity	Signalling
TRINITY_DN670774_c1_g1	0.384197633136154	0.0470588235294118	13	TRINITY_DN670774_c1_g1_i12	P83510	TNIK_MOUSE	9.88E-43	.	Signalling
TRINITY_DN655853_c2_g1	0.381622222223333	0.0470588235294118	15	TRINITY_DN655853_c2_g1_i14			.	.	
TRINITY_DN712378_c3_g1	0.369644444444167	0.0489887640449438	12	TRINITY_DN712378_c3_g1_i13	Q11212	ACT_SPOLI	5.79E-62	.	Actin: cytoskeleton
TRINITY_DN712378_c3_g1	0.369644444444167	0.0489887640449438	12	TRINITY_DN712378_c3_g1_i11	P10984	ACT2_CAEEL	1.54E-91	.	Actin: cytoskeleton
TRINITY_DN712378_c3_g1	0.369644444444167	0.0489887640449438	12	TRINITY_DN712378_c3_g1_i18	P48465	ACT_CRYNH	8.72E-22	.	Actin: cytoskeleton
TRINITY_DN712378_c3_g1	0.369644444444167	0.0489887640449438	12	TRINITY_DN712378_c3_g1_i16	P53464	ACTM_HELTB	2.27E-99	.	Actin: cytoskeleton
TRINITY_DN712378_c3_g1	0.369644444444167	0.0489887640449438	12	TRINITY_DN712378_c3_g1_i6	Q11212	ACT_SPOLI	5.1E-67	.	Actin: cytoskeleton
TRINITY_DN712378_c3_g1	0.369644444444167	0.0489887640449438	12	TRINITY_DN712378_c3_g1_i22	Q11212	ACT_SPOLI	1.01E-103	.	Actin: cytoskeleton
TRINITY_DN712378_c3_g1	0.369644444444167	0.0489887640449438	12	TRINITY_DN712378_c3_g1_i15	P10984	ACT2_CAEEL	3.2E-91	.	Actin: cytoskeleton
TRINITY_DN697274_c3_g2	0.369019526626577	0.0493333333333334	26	TRINITY_DN697274_c3_g2_i6			.	.	
TRINITY_DN728436_c0_g1	0.369108086785385	0.0493333333333334	13	TRINITY_DN728436_c0_g1_i8	Q96MR6	CFA57_HUMAN	2.55E-39	.	cilia protein
TRINITY_DN728436_c0_g1	0.369108086785385	0.0493333333333334	13	TRINITY_DN728436_c0_g1_i6	Q9D180	CFA57_MOUSE	2.62E-07	.	cilia protein
TRINITY_DN728436_c0_g1	0.369108086785385	0.0493333333333334	13	TRINITY_DN728436_c0_g1_i19	Q96MR6	CFA57_HUMAN	0	GO:0005515: protein binding	cilia protein
TRINITY_DN728436_c0_g1	0.369108086785385	0.0493333333333334	13	TRINITY_DN728436_c0_g1_i3	Q96MR6	CFA57_HUMAN	1.18E-46	.	cilia protein

Supplementary Table 4: Blast output from oligotype analyses. Oligotypes with superscript numerals indicate those which matched polar study accessions supported by the associated references in Supplementary references.

**Oligotype**	**Accession number of best match**	% identity	Description
[3]TT	HG941681	100	Uncultured *Granulosicoccus sp*
AC	LC171298	99.8	Uncultured beta proteobacterium
AA	EU290343	100	Uncultured bacterium
GG	EU290387	99.3	Uncultured bacterium
CT	JQ218768	99.6	Uncultured proteobacterium
[4]CG	GU235526	99.1	Uncultured marine bacterium
CA	KU647988	98.4	Uncultured bacterium
GC	HG934341	99.1	Uncultured *Leucothrix sp.*
TC	KC462967	99.1	*Shewanella sp.*
CC	EU290380	96.9	Uncultured bacterium
GT	EF466030	92.1	Uncultured episilon proteobacterium
AT	KJ615748	93.6	Uncultured bacterium
GA	GQ274134	97.8	Uncultured *Bacteriodetes*
[5]TA	GQ472830	97.4	Uncultured *Verrucomicrobia sp.*
AG	AY527410	98.9	*Saprospira sp.*
TG	LC171275	96.9	Uncultured *Bacteriodetes*

Supplementary references

1. Szklarczyk D, Gable AL, Lyon D, Junge A, Wyder S, Huerta-Cepas J, Simonovic M, Doncheva NT, Morris JH, Bork P, Jensen LJ, von Mering C. STRING v11: protein-protein association networks with increased coverage, supporting functional discovery in genome-wide experimental datasets. *Nucleic Acids Research*, **47**, D607-613 (2019)
2. Szklarczyk D, Morris JH, Cook H, Kuhn M, Wyder S, Simonovic M, Santos A, Doncheva NT, Roth A, Bork P, Jensen LJ, von Mering C. The STRING database in 2017: quality-controlled protein-protein association networks, made broadly accessible. *Nucleic Acids Research*, **45**, D362-68 (2017)
3. Cowie ROM, Williams GJ, Maas EW, Voyles KM, Ryan KG. Antarctic sea-ice microbial communities show distinct patterns of zonation in response to algal-derived substrates. *Aquatic Microbiology and Ecology*, **73**, 123-134 (2014)
4. Grzymski JJ, Riesenfeld CS, Williams TJ, Dussaq AM, Ducklow H, Erickson M, Cavicchioli R, Murray AE. A metagenomics assessment of winter and summer bacterioplankton from Antarctic Peninsula coastal surface waters. *ISME Journal*, **6**, 1901-1915 (2012)
5. Zeng Y, Zou Y, Grebmeier JM, He J, Zheng T. Culture-independent and –dependent methods to investigate the diversity of planktonic bacteria in the northern Bering Sea. *Polar Biology*, **35**, 117-129 (2012)
