# Supplementary material for: Lack of long-term acclimation in Antarctic encrusting species suggests vulnerability to warming
Source: Nat Commun. 2019 Jul 29;10:3383. doi: 10.1038/s41467-019-11348-w (PMC6662708; doi:10.1038/s41467-019-11348-w)
Supplement: Supplementary file 4 — Description of Additional Supplementary Files [file 41467_2019_11348_MOESM4_ESM.pdf]

Description of Additional Supplementary Files

File Name: Supplementary Data 1
Description: Transcripts up-regulated in animals on plus 2°C panels
